# Longitudinal Association Between Menopausal Transition and Obstructive Sleep Apnea with Effect Modification by Salt Intake: A Prospective Cohort Study

**DOI:** 10.3390/nu17223612

**Published:** 2025-11-19

**Authors:** Sujeong Shin, Yoonyoung Jang, Yoosoo Chang, Seungho Ryu

**Affiliations:** 1 Department of Family Medicine, Kangbuk Samsung Hospital, Sungkyunkwan University School of Medicine, Seoul 03181, Republic of Korea; 2 Center for Cohort Studies, Total Healthcare Center, Kangbuk Samsung Hospital, Sungkyunkwan University School of Medicine, Seoul 04514, Republic of Korea; 3 Institute of Medical Research, Sungkyunkwan University School of Medicine, Suwon 16419, Republic of Korea; 4 Department of Occupational and Environmental Medicine, Kangbuk Samsung Hospital, Sungkyunkwan University School of Medicine, Seoul 04514, Republic of Korea; 5 Department of Clinical Research Design & Evaluation, Samsung Advanced Institute for Health Sciences & Technology, Sungkyunkwan University, Seoul 06355, Republic of Korea

**Keywords:** dietary salt intake, menopausal transition, menopause, obstructive sleep apnea

## Abstract

**Background/Objectives**: As high salt intake may increase obstructive sleep apnea (OSA) risk through fluid retention and upper airway narrowing during sleep, we aimed to determine whether dietary salt intake modified the association between menopausal transition and the risk of OSA. **Methods**: Between 2014 and 2018, we conducted a longitudinal study of 2572 women aged 42–52 years at the Kangbuk Samsung Hospital Total Healthcare Center. The participants were followed up until the end of 2024. OSA risk was evaluated using the STOP-Bang questionnaire, with a body mass index threshold adjusted to ≥30 kg/m^2^ in accordance with a Korean validation study. Dietary salt intake was categorized into tertiles, with tertile 3 representing the highest salt intake. Generalized estimating equations with time-dependent covariates were used to account for repeated measurements over time. **Results**: OSA risk increased during menopausal transition. Compared with the pre-menopausal stage, both late transition (β = 0.41, 95% confidence interval [CI] 0.05–0.78) and post-menopause (β = 0.61, 95% CI 0.20–1.02) were significantly associated with an increased risk of OSA, independent of potential confounders. A high salt intake (tertile 3) was also significantly associated with OSA. A significant interaction was observed between menopausal transition and salt intake (*p* = 0.040), with a stronger association between menopausal transition and OSA during the early transition stage in women with higher salt intake. **Conclusions**: Menopausal transition and high dietary salt intake appear to act synergistically to increase the risk of OSA in middle-aged women. Our results suggest that implementing targeted screening and reducing dietary salt intake may mitigate the risk of OSA during menopausal transition.

## 1. Introduction

Obstructive sleep apnea (OSA) affects nearly one billion individuals worldwide, with a marked increase over the past 20–25 years [1,2,3]. OSA is strongly associated with a broad range of cardiovascular, metabolic, and neuropsychiatric issues and significantly increases morbidity across various organ systems. These extensive associations collectively impose a significant burden on quality of life [4,5,6]. While OSA has long been regarded as predominantly affecting men, it is now recognized as being substantially underdiagnosed in women. This under-recognition arises from their frequent presentation with non-classic symptoms, such as insomnia, fatigue, and mood disturbances, rather than classic loud snoring or observed apnea, ultimately obscuring the true burden of OSA in women [7,8].

This issue can become particularly relevant during menopausal transition, a phase characterized by a decline in estrogen and progesterone levels that can weaken upper airway muscle tone, disrupt ventilatory stability, and promote central fat accumulation. These changes increase the risk of sleep-disordered breathing [9,10] and emphasize the critical need to enhance the early identification and management of OSA in midlife women. In addition, high dietary salt intake has been proposed as another contributor to OSA, primarily by fostering fluid retention that may shift toward the head and neck during sleep, thereby narrowing the upper airway and increasing its collapsibility [11,12]. This mechanism is of particular concern in Korea, where the mean salt consumption significantly surpasses global dietary guidelines [13]. While menopausal transition has been recognized as a critical period for the emergence of sleep-disordered breathing, the combined effects of hormonal changes and dietary factors on OSA risk have not been well established. In particular, although salt intake has been implicated in OSA through mechanisms involving fluid retention and upper airway narrowing [11,12], its modifying role during the menopausal transition remains unclear. Given that salt consumption is highly prevalent yet modifiable in Asian populations, clarifying this relationship has important clinical and public health implications. Therefore, this study aimed to determine whether dietary salt intake modified the association between menopausal transition and OSA risk in a large cohort of middle-aged Korean women. We hypothesized that higher dietary salt intake would have synergistic effects with menopausal transition, thereby amplifying OSA risk and highlighting a modifiable target for the prevention of sleep-disordered breathing in this under-studied population.

## 2. Materials and Methods

### 2.1. Study Population

This retrospective longitudinal study recruited women aged 42–52 years between 2014 and 2018 who had undergone comprehensive health examinations at the Kangbuk Samsung Hospital Total Healthcare Center [14,15] and were followed until 2024. All participants provided written informed consent at enrollment for data collection aimed at understanding physical, physiological, and psychosocial changes during the menopausal transition. The original cohort project was approved by the Institutional Review Board (IRB) of Kangbuk Samsung Hospital (IRB Nos. 2014-01-081 and 2023-05-036). The present study was a secondary analysis using pre-existing, de-identified data from this consented cohort. Although the study objectives aligned with the cohort’s overall purpose, each secondary analysis underwent separate IRB review. The current study protocol was approved by the IRB of Kangbuk Samsung Hospital, which granted an exemption from additional consent requirements (IRB No. KBSMC 2025-07-026).

**Figure 1:**
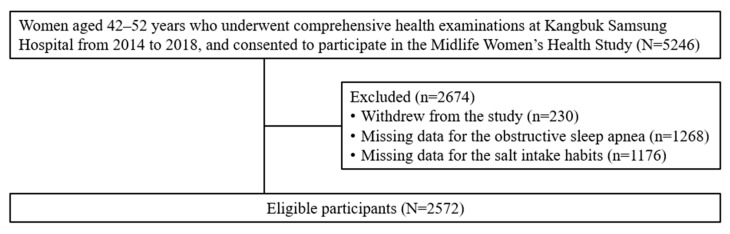
Flowchart of the participants.

Participant eligibility criteria have been reported in our previous studies [16,17]. The STOP-Bang questionnaire was introduced in 2018 to assess OSA. Of 5246 initial participants, we excluded 230 who withdrew from the study, 1268 with missing OSA data, and 1176 with no available information on salt intake habits. As the STOP-Bang and salt intake questionnaires were not part of the mandatory core assessments, some participants did not complete them. Subsequently, 2572 participants were included in the final analysis (Figure 1).

All study procedures have therefore been performed in accordance with the ethical standards laid down in the 1964 Declaration of Helsinki and its later amendments, as well as with the approved protocols and relevant regulations.

### 2.2. Measurement

Demographic and lifestyle characteristics, reproductive factors, and medical history were obtained using a self-administered standardized questionnaire. Smoking status was classified as ever smoker (including both former and current smokers) for individuals with a lifetime consumption of more than five packs and as never smoker otherwise [18]. Alcohol consumption was dichotomized based on an average daily intake of 10 g [19]. Physical activity was assessed using the Korean version of the short-form International Physical Activity Questionnaire and categorized as inactive, moderately active, or health-enhancing physical activity (HEPA) [20,21]. Age at menarche was categorized as <12, 12–13, 14–16, and ≥17 years. Parity was categorized as nulliparous or parous. Marital status was classified as married/cohabiting, unmarried, or divorced/separated/widowed. A history of diabetes mellitus [22] and the use of medication for hyperlipidemia were dichotomized as either yes or no.

### 2.3. Definition of Menopausal Stages

According to Stages of Reproductive Aging Workshop (STRAW)+10 criteria [23], menopausal stages were classified into four categories. The pre-menopausal stage was defined as regular menstrual cycles that did not meet any of the criteria for the subsequent stages. The early transition stage was defined as having at least two menstrual cycles with a difference of ≥7 days. The late transition stage was defined as the presence of amenorrhea for ≥60 days. The post-menopausal stage was defined as no menstruation for ≥12 months.

### 2.4. Assessment of Salt Intake Habits

Salt intake habits were assessed using a self-reported questionnaire comprising the following three items [24,25]: (1) “What are your salt-related taste preferences?” (very salty = 12, slightly salty = 9, modestly salty = 6, slightly bland = 3, very bland = 0); (2) “Do you add salt or soy sauce to cooked dishes?” (always = 12, frequently = 8, seldom = 4, never = 0), and (3) “Do you eat pan-fried or deep-fried food with soy sauce?” (always = 12; sometimes = 6; never = 0). The total salt-intake habit score was calculated by summing the scores of the three items, yielding a possible range from 0 to 36.

In a previous validation study of the salt intake habit score conducted among 189 normotensive Korean adults [25], the summed score was significantly correlated with 24-h urinary sodium (partial Spearman r = 0.26, *p* < 0.001) after adjustment for age, sex, and BMI.

The salt intake score was non-normally distributed (Shapiro–Wilk test, *p* < 0.001). Tertiles were defined using baseline cut-off values of 10 and 16 points, which were consistently applied to all repeated measurements during follow-up. The upper cutoff (16 points) corresponded to the median value reported for women in the original validation study [25], and was selected to ensure adequate group sizes given the low prevalence of OSA and absence of established clinical thresholds. For the analysis, participants were dichotomized into two groups (tertiles 1–2 vs. tertile 3). Salt intake was treated as a time-varying variable in the GEE models, allowing dietary habits to change over time while maintaining consistent categorization criteria. For the analysis, participants were grouped into tertiles 1–2 and tertile 3.

### 2.5. Definition of OSA

OSA was evaluated using a modified version of the STOP-Bang questionnaire [26], in which the body mass index (BMI) cut-off was lowered from 35 kg/m^2^ to 30 kg/m^2^ to reflect thresholds more appropriate for Asian populations [27]. This modification is supported by validation studies demonstrating comparable or improved predictive performance of the STOP-Bang questionnaire using a BMI threshold of 30 kg/m^2^ in Korean and other East Asian populations [27,28,29,30].

The STOP-Bang consists of eight items: four STOP items—snoring, tiredness, observed apnea, and presence of hypertension—and four Bang items—BMI ≥ 30 kg/m^2^ [27,31], age ≥ 50 years, neck circumference ≥ 41 cm, and male sex.

Snoring was assessed by asking, “Do you snore loudly (loud enough to be heard through closed doors or your bed partner elbows you for snoring at night)?” Tiredness was evaluated using the following question: “Do you often feel tired, fatigued, or sleepy during the daytime (such as when driving)?” Observed apnea was assessed using the following question: “Has anyone observed you stop breathing or choking/gasping during your sleep?” Large neck circumference was assessed using the question, “Is your neck size large (defined as a circumference greater than 41 cm in females, measured around the laryngeal prominence)?” [26]. Hypertension was defined as involving at least one of the following: systolic blood pressure ≥ 140 mmHg, diastolic blood pressure ≥ 90 mmHg, or current use of blood pressure-lowering medication [32]. BMI was calculated from the measured height and weight. Data concerning age and sex were obtained from participant demographics recorded at the time of hospital registration, which are routinely verified in Korea using the resident registration number.

Each item was scored as 1 if present and 0 if absent, yielding a total score ranging from 0 to 8 [26]. Total scores were categorized as follows: 0–2, low risk; 3–4, intermediate risk; ≥5, high risk [26]. In addition, participants with a total score of 2–4 were also classified as high risk if they met either of the following criteria [26]: BMI, ≥30 kg/m^2^ [31] or neck circumference, ≥41 cm.

### 2.6. Statistical Analyses

Baseline characteristics were summarized using means and standard deviations for continuous variables and frequencies and proportions for categorical variables. Because this study utilized repeated measures from 2020 to 2024, we employed a generalized estimating equation (GEE) model to account for within-subject correlations [33]. The model specifies an exchangeable working correlation structure with individual identifiers as the clustering variables. A binomial logistic link function and a robust standard error were applied.

To evaluate model fit in the GEE analysis, we compared the quasi-likelihood under the independence model criterion (QIC) across correlation structures: exchangeable (QIC = 2742) and independent (QIC = 2755). The exchangeable structure was selected based on the lower QIC value. The autoregressive correlation structure was not considered, as it is more appropriate for data with equally spaced follow-up intervals between repeated measures [34], whereas our longitudinal cohort was based on routine health examinations with irregular observation intervals across individuals. As part of the sensitivity analysis, an independent correlation structure was applied and compared with the primary exchangeable model.

Since completion of the STOP-Bang questionnaire was optional, analyses were restricted to participants who provided responses. Although the cohort was initially recruited between 2014 and 2018 and followed through 2024, the STOP-Bang questionnaire was introduced in 2018 as a nonmandatory item. Therefore, responses varied depending on when participants first completed the questionnaire between 2018 and 2024. Given the study’s objective to evaluate the association between menopausal transition and OSA risk, the first available STOP-Bang response was treated as the baseline assessment, and follow-up was defined accordingly. Among the final analytic sample of 2572 women, 43.4% were observed only once (with the remaining participants observed on two or more occasions). As some participants contributed data from a single observation, inverse probability weighting (IPW) was applied to address potential selection bias related to observation frequency [35]. For each participant, we estimated the probability of being observed on two or more occasions (versus once) using a logistic regression model, with observation status (two or more = 1, once = 0) as the dependent variable and baseline characteristics—age, menopausal stage, smoking status, alcohol consumption, physical activity, BMI, age at menarche, parity, marital status, and educational attainment—as independent variables. Individual weights were derived as the inverse of the predicted probability of each participant’s observation status and incorporated into the GEE models as an additional sensitivity analysis to account for potential selection bias.

The primary exposures were menopausal transition and salt intake, including their interaction terms. The outcome variable was OSA risk (low vs. intermediate/high). The model was adjusted for age (years), smoking status, alcohol consumption, physical activity level, BMI (continuous), parity, marital status, and education level as time-varying covariates, and for age at menarche as a fixed covariate. Missing data were observed only in four categorical variables (education level, marital status, parity, and smoking status), with missing rates below 2.0%. These categorical variables were automatically dummy-coded using Stata’s factor-variable notation (i. prefix), which generates indicator variables for each category within a model. The significance of the interaction terms was evaluated using Wald’s test. Statistical significance was defined as a two-sided *p*-value < 0.05. All analyses were performed using STATA, version 18 (StataCorp LLC, College Station, TX, USA).

## 3. Results

The study participants’ baseline characteristics are presented in Table 1. Among the 2572 women included in the analysis, the prevalence of each STOP-Bang component at baseline was as follows: snoring (11.7%), tiredness (53.4%), observed apnea (2.2%), presence of hypertension (6.2%), BMI ≥ 30.0 kg/m^2^ (2.2%), age ≥ 50 years (33.6%), and large neck circumference ≥ 41 cm (3.1%). Given that all the participants were women, the sex item in the STOP-Bang questionnaire—where male sex is considered a risk factor—was coded as “no” across the entire sample. During the study period (2018–2024), participants had a median of 2 visits (interquartile range, 1–3), with a maximum of 8 visits. For repeated measurements, the median interval between consecutive visits was 1.1 years (interquartile range, 0.9–2.0 years).

After adjusting for smoking status, alcohol consumption, physical activity level, BMI (continuous), parity, marital status, education level, and age at menarche, the late transition stage (β = 0.41, 95% confidence interval [CI] 0.05–0.78) and the post-menopausal stage (β = 0.61, 95% CI 0.20–1.02) were significantly associated with OSA, compared with the pre-menopausal stage. No significant association was observed for the early transition stage. Additionally, time-varying age was associated with a higher likelihood of developing OSA (β = 0.21, 95% CI 0.17–0.25). A high salt intake (tertile 3) was significantly associated with OSA (β = 0.41, 95% CI 0.18–0.65) (Supplementary Table S1). The other covariates were not significantly associated with OSA (Supplementary Table S2).

**Figure 2:**
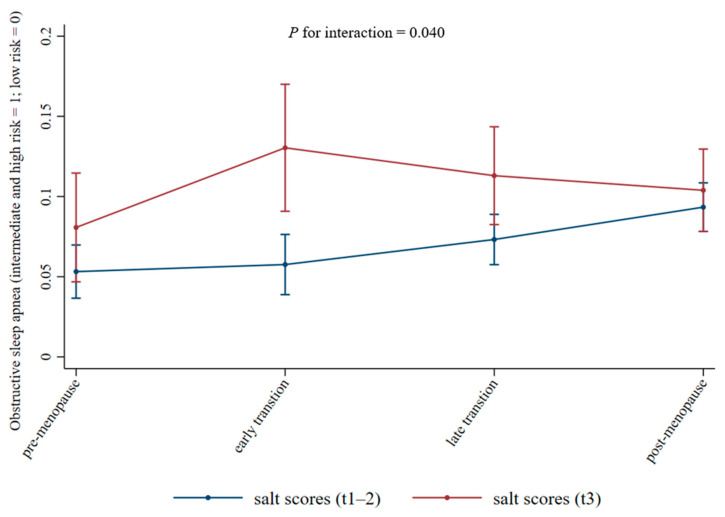
Trends in the risk of obstructive sleep apnea across menopausal transition stages, stratified by salt intake. Error bars represent 95% confidence intervals.

The association between menopausal transition and OSA differed in accordance with salt intake level (tertiles 1–2 vs. tertile 3), with a significant interaction (*p* = 0.040, Figure 2). In the highest tertile of salt intake, OSA risk significantly increased during the early transition stage (β = 0.64, 95% CI 0.02–1.27), suggesting a potential increase in OSA risk. In contrast, among women with lower salt intake (tertiles 1–2), OSA risk became evident during the post-menopausal stage (β = 0.72, 95% CI 0.26–1.17), after adjusting for the same covariates (Table 2).

A sensitivity analysis using the independent correlation structure yielded results consistent in direction and overall pattern with the main exchangeable model. Although the interaction term between menopausal status and salt intake became marginally significant (*p* for interaction = 0.061) under the independent structure, the overall interpretation remained unchanged, supporting the robustness of the findings (Supplementary Figure S1). An additional sensitivity analysis incorporating IPW also produced results consistent with the primary analysis (*p* for interaction = 0.039) (Supplementary Figure S2).

## 4. Discussion

In this large cohort of middle-aged Korean women, we observed a progressive increase in the risk of OSA across menopausal transition stages, and the association became significant from the late transition stage onward, as assessed using the STOP-Bang questionnaire. Dietary salt intake emerged as an independent determinant of OSA risk. Women with a lower salt intake exhibited a relatively low risk of OSA during both the early and late transition stages, with the risk becoming evident only during the post-menopausal stage. In contrast, women with higher salt consumption showed a higher baseline risk and a markedly steeper increase in OSA risk beginning at the early transition stage. This risk appeared to attenuate slightly in the later stages; however, it remained consistently higher than that for women with lower salt intake. These findings suggest that salt intake may act synergistically with hormonal changes, particularly during the early menopausal transition, to exacerbate vulnerability to sleep-disordered breathing in middle-aged women.

These findings are particularly relevant because OSA in women is often under-recognized owing to its atypical symptom presentation [36]. Compared with men, women more frequently report insomnia, fatigue, or mood disturbances than the classic features of loud snoring or excessive daytime sleepiness [7,37]. This under-diagnosis has important clinical implications, as untreated OSA is associated with cardiovascular and metabolic complications that may disproportionately affect women during midlife [3,4,5]. In our cohort, typical symptoms of OSA, such as loud snoring or observed apnea, were infrequent. Conversely, fatigue was identified as the most frequently reported symptom, corresponding to the typical manifestation of OSA in women. Therefore, an accurate assessment of OSA risk is essential for women, which prompted us to apply the STOP-Bang questionnaire in this study.

Specifically, we used the STOP-Bang questionnaire to assess OSA risk given its feasibility in large-scale epidemiological settings and its validated predictive utility, as demonstrated by significant correlations with polysomnographic indices such as the apnea-hypopnea index in numerous studies [26,31,38,39]. In our study, we applied a BMI threshold of ≥30 kg/m^2^ in relation to the STOP-Bang questionnaire. Previous validation studies have shown little difference in predictive performance when using BMI cut-offs of 30 versus 35 kg/m^2^ [27,28]. A Korean study reported that adopting a threshold of 30 kg/m^2^ improved both the sensitivity and specificity for detecting OSA, supporting the appropriateness of this criterion in our cohort [29,30]. Specifically, we defined high-risk OSA as a STOP-Bang score ≥ 3, a threshold validated in prior studies. While this cut-off lowers specificity, it enhances sensitivity and is particularly suitable for under-diagnosed populations, such as women [26,38]. This criterion was applied in our study and is consistent with the current stepwise screening recommendations. This approach was appropriate for identifying high-risk individuals in this large-scale population-based study, particularly when full polysomnography was not feasible.

The effect of salt intake observed in this study is biologically plausible. High dietary salt expands extracellular fluid volume and promotes nocturnal rostral fluid shifts, leading to peripharyngeal edema and increased upper-airway collapsibility [11,40]. A recent prospective cohort study supported this finding, reporting that the practice of adding salt to meals is independently linked to a higher occurrence of OSA [11]. Excess salt also elevates blood pressure and activates the renin–angiotensin–aldosterone system and sympathetic pathways, which destabilize ventilatory control [41,42]. Elevated blood pressure may, in turn, exacerbate OSA by promoting vascular congestion and peripharyngeal fluid accumulation [43]. At the same time, OSA can aggravate hypertension through intermittent hypoxia and sympathetic overactivity, creating a reciprocal interaction between the two conditions [44]. The temporal relationship between salt intake and OSA risk varies across the menopausal stages, highlighting distinct vulnerability windows. During perimenopause, including early and late transition stages, fluctuating and declining estrogen disrupts RAAS regulation, increasing aldosterone sensitivity and epithelial sodium channel (ENaC) activity in the upper airway, while concurrently declining progesterone reduces ventilatory drive and genioglossus muscle responsiveness [38,45]. High salt intake during this transitional phase may further exacerbate fluid retention and nocturnal rostral fluid shift, amplifying upper airway collapsibility―making perimenopause a critical window for preventive sodium reduction. In the postmenopausal stage, OSA risk was elevated even in low salt intake, reflecting the cumulative impact of hormonal decline, central adiposity, airway structural changes, and chronic RAAS dysregulation [46]. These multifactorial changes may attenuate the protective effect of sodium restriction alone, suggesting that integrated strategies—combining salt reduction with weight management, physical activity, and potential RAAS or hormonal modulation—are likely required to effectively mitigate OSA risk in this population [9]. Future longitudinal studies should evaluate the efficacy of stage-specific interventions to guide optimal timing and personalization of preventive approaches.

To date, few studies have jointly examined the association between dietary salt intake and menopausal transition and the risk of OSA. Our findings address this gap by showing that the adverse effects of high salt intake were most evident during early menopausal transition, underscoring this stage as a critical window for preventive interventions. The influence of salt intake appears to diminish during the later transition and post-menopausal stages. One possible explanation is that women with a long-standing high salt intake may develop hypertension and initiate treatment, thereby attenuating this association [47]. Hormonal changes during menopausal transition, particularly estrogen decline, also contribute to rising blood pressure and may further influence this association [48]. However, our data did not allow us to confirm this pathway. Further studies that incorporate longitudinal blood pressure trajectories and antihypertensive medication use are required to clarify these mechanisms.

This study had some limitations. Data concerning menopausal stage and salt intake habits were collected using a self-reported questionnaire. Despite employing a validated questionnaire to ensure the reliability of the results [24,49], no further verification using objective biomarkers to assess the menopausal stage and salt intake habits was conducted. Survey-based assessments of dietary intakes are inherently subject to measurement error [50]. For sodium intake specifically, correlations with 24-h urinary sodium—the gold standard—are generally low, with correlation coefficients ranging from 0.15 to 0.30 [25,51], reflecting the substantial influence of recall bias and social-desirability bias on self-reported dietary behaviors [52]. However, since our study examined whether salt intake modified the association between menopausal transition and OSA risk, there is no plausible reason why reporting error would differ by menopausal stage or OSA status. Therefore, any misclassification of salt intake is likely non-differential, which would attenuate associations toward the null, suggesting that our findings may underestimate the true modifying effect [53]. Further studies using objectively measured data to quantify menopausal status and habitual salt intake are required to confirm these findings. OSA risk was assessed using a screening tool rather than polysomnography. However, the STOP-Bang questionnaire has been widely validated and is appropriate for large-scale risk evaluations [39,54,55]. This approach also reflects clinical practice in which risk assessment, particularly in peri- and post-menopausal women, guides decisions regarding further diagnostic testing. Future studies that incorporate objective diagnostic measures are necessary to confirm these associations. Multiple covariates were included in the regression models, but residual confounding or unmeasured variables may exist. However, we applied GEEs with time-dependent covariates to account for the temporal changes in exposure and covariates during the follow-up period. The study participants were generally well educated and experienced fewer financial difficulties than the broader population [15]. Additional studies are necessary to replicate these results and assess their applicability in middle-aged Korean women. Finally, our study population exclusively consisted of Korean women, which may limit its generalizability to other ethnicities or cultural groups.

## 5. Conclusions

This longitudinal cohort study examined the association between menopausal transition, dietary salt intake, and OSA risk in a large cohort of middle-aged Korean women. The OSA risk increased with advancing menopausal stage and was further elevated in women with higher salt intake. The association between menopausal transition and OSA risk varied according to the level of dietary salt intake, suggesting that salt intake may amplify vulnerability to OSA during this period. Among women with lower salt intake, the baseline OSA risk was relatively low and increased modestly across the menopausal stages. In contrast, those with a higher salt intake exhibited both a higher baseline risk and a more pronounced increase in OSA risk, particularly during the early menopausal transition. These findings suggest that targeted screening for OSA during the menopausal transition and dietary salt reduction may help mitigate the risk of sleep-disordered breathing in middle-aged women. Given the heightened susceptibility to sodium excess and sodium-induced fluid retention during the menopausal transition, integrating salt intake assessment into routine menopausal health evaluations could provide a critical opportunity for OSA prevention. Dietary sodium reduction as part of lifestyle counseling is a feasible, cost-effective, and evidence-based strategy that aligns with cardiovascular preventive guidelines, offering dual benefits for cardiometabolic and sleep health during this vulnerable period. Future studies should explore the causal factors underlying these associations and evaluate how salt reduction strategies influence OSA risk across different stages of menopausal transition.

These findings suggest that targeted screening for OSA during the menopausal transition and dietary salt reduction may help mitigate the risk of sleep-disordered breathing in middle-aged women. Future studies should explore the causal factors underlying these associations and evaluate how salt reduction strategies affect the risk of OSA during different phases of menopausal transition.

## Figures and Tables

**Table 1:** Baseline characteristics (N = 2572).

Variable	Frequency (%)
Age at baseline ^1^	48.3 ± 3.5
Risk factors for OSA	
Snoring	300 (11.7)
Tiredness	1373 (53.4)
Observed apnea	57 (2.2)
Presence of hypertension	160 (6.2)
BMI (≥30.0 kg/m^2^)	57 (2.2)
Age (≥50 years)	871 (33.6)
Neck circumference (≥41 cm)	79 (3.1)
Menopausal stages	
Pre-menopause	942 (36.6)
Early transition	515 (20.0)
Late transition	500 (19.4)
Post-menopause	615 (23.9)
Age at menarche	
<12 years	114 (4.4)
12–13 years	922 (35.9)
14–16 years	1446 (56.2)
≥17 years	90 (3.5)
Smoking	
Never	2235 (86.9)
Current/Former	323 (12.6)
Unknown	14 (0.5)
Alcohol consumption	
<10 g/day	2404 (93.5)
≥10 g/day	168 (6.5)
Parity	
Nulliparous	186 (7.2)
Parous	2340 (91.0)
Unknown	46 (1.8)
Marital status	
Married/co-habiting	2408 (93.6)
Unmarried	101 (3.9)
Divorced/Separated/Widowed	63 (2.5)
Education	
≤High school	473 (18.4)
≥College	2060 (80.1)
Unknown	39 (1.5)
Physical activity ^2^	
Inactivity	872 (33.9)
Moderate activity	1259 (49.0)
HEPA	441 (17.2)
History of diabetes mellitus ^3^	
Yes	74 (2.9)
Medication for hyperlipidemia	
Yes	112 (4.3)

^1^ Baseline age is presented as the mean and standard deviation (SD); ^2^ Physical activity was assessed by using the Korean version short form of the International Physical Activity Questionnaire; ^3^ Diabetes mellitus, defined as a fasting plasma glucose level ≥ 126 mg/dL, a hemoglobin A1c (HbA1c) level ≥ 6.5%, or antidiabetic medication use; Abbreviations: BMI, body mass index; HEPA, health-enhancing physical activity; OSA, obstructive sleep apnea.

**Table 2:** Associations between menopausal transition and OSA stratified according to salt intake scores.

Variable	Coefficient (95% CI)		*p* for Interaction
	Salt Intake Scores (Tertiles 1–2)	Salt Intake Scores (Tertile 3)	
Menopausal transition			0.040
Pre-menopause	Ref	Ref	
Early transition	0.10 (−0.38–0.58)	0.64 (0.02–1.27)	
Late transition	0.40 (−0.02–0.82)	0.45 (−0.19–1.08)	
Post-menopause	0.72 (0.26–1.17)	0.33 (−0.32–0.98)	

The model was adjusted for age (year), smoking status (never smoker, ever smoker, unknown), alcohol consumption (<10 g/day, ≥10 g/day, unknown), physical activity level (inactive, moderately active, HEPA, unknown), BMI (continuous), parity (nulliparous, parous, unknown), marital status (unmarried, married/cohabiting, divorced/separated/widowed, unknown), and education level (high school graduate or less, college graduate or higher, unknown) as time-varying covariates; and for age at menarche (<12, 12–13, 14–16, ≥17 years, unknown) as a fixed covariate, with clustering according to individual identifiers. Abbreviations: BMI, body mass index; CI, confidence.

## Data Availability

The data supporting the findings of this study are not publicly available at present, but the analytical methods and dataset are available from the corresponding author upon request.

## Supplementary Materials

The following supporting information can be downloaded at: https://www.mdpi.com/article/10.3390/nu17223612/s1, Figure S1: Sensitivity analysis of OSA risk trends across menopausal transition stages by salt intake (independent correlation). Error bars represent 95% confidence intervals; Figure S2: Sensitivity analysis of OSA risk trends across menopausal transition stages by salt intake, incorporating inverse probability weighting (IPW); Table S1: Association between menopausal transition and salt intake and OSA; Table S2: Associations between OSA and covariates.

## Abbreviations

The following abbreviations are used in this manuscript:

BMI	body mass index
CI	confidence interval
GEE	generalized estimating equation
HEPA	health-enhancing physical activity
NIH	National Institute of Health
OSA	Obstructive sleep apnea
STRAW	Stages of Reproductive Aging Workshop

## References

[1] Dantas A.B.d.A., Gonçalves F.M., Martins A.A., Alves G.Â., Stechman-Neto J., Corrêa C.d.C., Santos R.S., Nascimento W.V., de Araujo C.M., Taveira K.V.M. (2023). Worldwide prevalence and associated risk factors of obstructive sleep apnea: A meta-analysis and meta-regression. Sleep Breath.

[2] Benjafield A.V., Ayas N.T., Eastwood P.R., Heinzer R., Ip M.S.M., Morrell M.J., Nunez C.M., Patel S.R., Penzel T., Pépin J.-L. (2019). Estimation of the global prevalence and burden of obstructive sleep apnoea: A literature-based analysis. Lancet Respir. Med..

[3] Cunningham J., Hunter M., Budgeon C., Murray K., Knuiman M., Hui J., Hillman D., Singh B., James A. (2021). The prevalence and comorbidities of obstructive sleep apnea in middle-aged men and women: The Busselton Healthy Ageing Study. J. Clin. Sleep Med..

[4] Strenth C., Wani A., Alla R., Khan S., Schneider F.D., Thakur B. (2024). Obstructive Sleep Apnea and Its Cardiac Implications in the United States: An Age-Stratified Analysis Between Young and Older Adults. J. Am. Heart Assoc..

[5] Lal C., Ayappa I., Ayas N., Beaudin A.E., Hoyos C., Kushida C.A., Kaminska M., Mullins A., Naismith S.L., Osorio R.S. (2022). The link between obstructive sleep apnea and neurocognitive impairment: An official American thoracic society workshop report. Ann. Am. Thorac. Soc..

[6] Garbarino S., Bardwell W.A., Guglielmi O., Chiorri C., Bonanni E., Magnavita N. (2020). Association of anxiety and depression in obstructive sleep apnea patients: A systematic review and meta-analysis. Behav. Sleep Med..

[7] BaHammam A.S. (2025). The gender gap in obstructive sleep apnea: Unmasking the disproportionate costs on women. Sleep.

[8] Bouloukaki I., Tsiligianni I., Schiza S. (2021). Evaluation of obstructive sleep apnea in female patients in primary care: Time for improvement?. Med. Princ. Pract..

[9] Zhou P., Li H., Li H., Chen Y., Lv Y. (2025). A possible important regulatory role of estrogen in obstructive sleep apnea hypoventilation syndrome. Front. Med..

[10] Perger E., Mattaliano P., Lombardi C. (2019). Menopause and sleep apnea. Maturitas.

[11] Li T., Song L., Li G., Li F., Wang X., Chen L., Rong S., Zhang L. (2023). Eating habit of adding salt to foods and incident sleep apnea: A prospective cohort study. Respir. Res..

[12] Fiori C.Z., Martinez D., Montanari C.C., Lopez P., Camargo R., Sezerá L., Gonçalves S.C., Fuchs F.D. (2018). Diuretic or sodium-restricted diet for obstructive sleep apnea—A randomized trial. Sleep.

[13] Han Y.-J., Jang E.-H., Lee S. (2023). Sodium intake trend and current intake level by meal provision place among the citizens of Seoul. Nutr. Res. Pract..

[14] Choi H.R., Chang Y., Park J., Cho Y., Kim C., Kwon M.-J., Kang J., Kwon R., Lim G.-Y., Ahn J. (2024). Early-onset vasomotor symptoms and development of depressive symptoms among premenopausal women. J. Affect. Disord..

[15] Namgoung S., Chang Y., Woo C., Kim Y., Kang J., Kwon R., Lim G., Choi H.R., Kim K., Kim H. (2022). Metabolically healthy and unhealthy obesity and risk of vasomotor symptoms in premenopausal women: Cross-sectional and cohort studies. BJOG Int. J. Obstet. Gynaecol..

[16] Jang Y., Chang Y., Park J., Kim C., Jeon S.W., Kang J., Kwon R., Lim G.-Y., Kim K.-H., Kim H. (2025). Menopausal stage transitions and their associations with overall and individual sleep quality in middle-aged Korean women. J. Affect. Disord..

[17] Park J., Chang Y., Choi H.R., Kim J.H., Seo S.W., Ryu H.J., Cho Y., Kim C., Kwon R., Lim G.-Y. (2024). Overactive bladder and cognitive impairment in middle-aged women: A cross-sectional study. Maturitas.

[18] Agaku I.T., King B.A., Dube S.R. (2014). Current cigarette smoking among adults—United States, 2005–2012. MMWR Morb. Mortal. Wkly. Rep..

[19] Liu Y., Colditz G.A., Rosner B., Berkey C.S., Collins L.C., Schnitt S.J., Connolly J.L., Chen W.Y., Willett W.C., Tamimi R.M. (2013). Alcohol intake between menarche and first pregnancy: A prospective study of breast cancer risk. J. Natl. Cancer Inst..

[20] Chun M.Y. (2012). Validity and reliability of Korean version of international physical activity questionnaire short form in the elderly. Korean J. Fam. Med..

[21] Oh J.Y., Yang Y.J., Kim B., Kang J.-H. (2007). Validity and reliability of Korean version of International Physical Activity Questionnaire (IPAQ) short form. Korean J. Fam. Med..

[22] Park S.E., Ko S.-H., Kim J.Y., Kim K., Moon J.H., Kim N.H., Han K.D., Choi S.H., Cha B.S. (2025). Diabetes fact sheets in Korea 2024. Diabetes Metab. J..

[23] Harlow S.D., Gass M., Hall J.E., Lobo R., Maki P., Rebar R.W., Sherman S., Sluss P.M., de Villiers T.J. (2012). Executive summary of the Stages of Reproductive Aging Workshop + 10: Addressing the unfinished agenda of staging reproductive aging. J. Clin. Endocrinol. Metab..

[24] Choi Y., Lee J.E., Chang Y., Kim M.K., Sung E., Shin H., Ryu S. (2016). Dietary sodium and potassium intake in relation to non-alcoholic fatty liver disease. Br. J. Nutr..

[25] Kim H.J., Paik H.Y., Lee S.Y., Shim J.E., Kim Y.S. (2007). Salt usage behaviors are related to urinary sodium excretion in normotensive Korean adults. Asia Pac. J. Clin. Nutr..

[26] Chung F., Abdullah H.R., Liao P. (2016). STOP-Bang questionnaire: A practical approach to screen for obstructive sleep apnea. Chest.

[27] Ong T.H., Raudha S., Fook-Chong S., Lew N., Hsu A.A.L. (2010). Simplifying STOP-BANG: Use of a simple questionnaire to screen for OSA in an Asian population. Sleep Breath.

[28] Vana K.D., Silva G.E., Goldberg R. (2013). Predictive abilities of the STOP-Bang and Epworth Sleepiness Scale in identifying sleep clinic patients at high risk for obstructive sleep apnea. Res. Nurs. Health.

[29] Byun J.-I., Kim D.-H., Kim J.-S., Shin W.C. (2020). Usefulness of using alternative body-mass index and neck circumference criteria for STOP-Bang questionnaire in screening South Korean obstructive sleep apnea patients. Sleep Med. Res..

[30] Kim B., Lee E.M., Chung Y.-S., Kim W.-S., Lee S.-A. (2015). The utility of three screening questionnaires for obstructive sleep apnea in a sleep clinic setting. Yonsei Med. J..

[31] Pavarangkul T., Jungtrakul T., Chaobangprom P., Nitiwatthana L., Jongkumchok W., Morrakotkhiew W., Kachenchart S., Chindaprasirt J., Limpawattana P., Srisaenpang S. (2016). The Stop-Bang Questionnaire as a Screening Tool for obstructive sleep apnea-induced hypertension in Asian population. Neurol. Int..

[32] Kim H.C., Lee H., Lee H.-H., Ahn S.V., Lee J.-M., Cheon D.Y., Jhee J.H., Yoon M., Shin M.-H., Heo J. (2025). Korea hypertension fact sheet 2024: Nationwide population-based analysis with a focus on young adults. Clin. Hypertens..

[33] Zorn C.J. (2001). Generalized estimating equation models for correlated data: A review with applications. Am. J. Political Sci..

[34] Bai Y., Huang J., Li R., You J. (2015). Semiparametric longitudinal model with irregular time autoregressive error process. Stat. Sin..

[35] Chesnaye N.C., Stel V.S., Tripepi G., Dekker F.W., Fu E.L., Zoccali C., Jager K.J. (2022). An introduction to inverse probability of treatment weighting in observational research. Clin. Kidney J..

[36] Bonsignore M.R., Saaresranta T., Riha R.L. (2019). Sex differences in obstructive sleep apnoea. Eur. Respir. Rev..

[37] Geer J.H., Hilbert J. (2021). Gender Issues in Obstructive Sleep Apnea. Yale J. Biol. Med..

[38] Chen L., Pivetta B., Nagappa M., Saripella A., Islam S., Englesakis M., Chung F. (2021). Validation of the STOP-Bang questionnaire for screening of obstructive sleep apnea in the general population and commercial drivers: A systematic review and meta-analysis. Sleep Breath.

[39] Cho T., Yan E., Chung F. (2024). The STOP-Bang questionnaire: A narrative review on its utilization in different populations and settings. Sleep Med. Rev..

[40] White L.H., Bradley T.D. (2013). Bradley, Role of nocturnal rostral fluid shift in the pathogenesis of obstructive and central sleep apnoea. J. Physiol..

[41] Kazi R.N.A. (2025). Silent Effects of High Salt: Risks Beyond Hypertension and Body’s Adaptation to High Salt. Biomedicines.

[42] Anderson D.E., Parsons B.A., McNeely J.D., Miller E.R. (2007). Salt sensitivity of blood pressure is accompanied by slow respiratory rate: Results of a clinical feeding study. J. Am. Soc. Hypertens..

[43] Balcan B., Akdeniz B., Peker Y., Collaborators T.T. (2024). Obstructive Sleep Apnea and Pulmonary Hypertension: A Chicken-and-Egg Relationship. J. Clin. Med..

[44] Brown J., Yazdi F., Jodari-Karimi M., Owen J.G., Reisin E. (2022). Obstructive Sleep Apnea and Hypertension: Updates to a Critical Relationship. Curr. Hypertens. Rep..

[45] Saaresranta T., Anttalainen U., Polo O. (2015). Sleep disordered breathing: Is it different for females?. ERJ Open Res..

[46] Moccia P., Belda-Montesinos R., Monllor-Tormos A., Chedraui P., Cano A. (2021). Body weight and fat mass across the menopausal transition: Hormonal modulators. Gynecol. Endocrinol..

[47] Grillo A., Salvi L., Coruzzi P., Salvi P., Parati G. (2019). Sodium intake and hypertension. Nutrients.

[48] El Khoudary S.R., Aggarwal B., Beckie T.M., Hodis H.N., Johnson A.E., Langer R.D., Limacher M.C., Manson J.E., Stefanick M.L., Allison M.A. (2020). Menopause transition and cardiovascular disease risk: Implications for timing of early prevention: A scientific statement from the American Heart Association. Circulation.

[49] Shen W., Cai L., Wang B., Li J., Sun Y., Chen Y., Xia F., Wang N., Lu Y. (2024). Associations of a proinflammatory diet, habitual salt intake, and the onset of type 2 diabetes: A prospective cohort study from the UK Biobank. Diabetes Obes. Metab..

[50] Shaw P.A., Deffner V., Keogh R.H., Tooze J.A., Dodd K.W., Küchenhoff H., Kipnis V., Freedman L.S. (2018). Epidemiologic analyses with error-prone exposures: Review of current practice and recommendations. Ann. Epidemiol..

[51] Freedman L.S., Commins J.M., Moler J.E., Arab L., Baer D.J., Kipnis V., Midthune D., Moshfegh A.J., Neuhouser M.L., Prentice R.L. (2014). Pooled results from 5 validation studies of dietary self-report instruments using recovery biomarkers for energy and protein intake. Am. J. Epidemiol..

[52] Soh Y.C., Fairley A., Alawad M., Lee S.S., Su T.T., Stephan B.C.M., Reidpath D., Robinson L., Yasin S., Siervo M. (2024). Assessing sodium intake in middle-aged and older adults with elevated blood pressure: Validation of spot urine excretion and dietary survey-derived estimates. Nutrients.

[53] Stayner L., Pearce N., Nøhr E.A., Freeman L.B., Deffner V., Ferrari P., Freedman L.S., Kogevinas M., Kromhout H., Lewis S. (2024). Information bias: Misclassification and mismeasurement of exposure and outcome. Statistical Methods in Cancer Research Volume V: Bias Assessment in Case–Control and Cohort Studies for Hazard Identification.

[54] Lee M.-K., Choi J.H., Lee J.Y. (2024). Validity of Modified STOP-Bang Questionnaire as a Screening Tool for Obstructive Sleep Apnea. Ann. Otol. Rhinol. Laryngol..

[55] Wang L., Zhao W., Liang C., Yan X., Zhang H., Dai H., Yu H., Zhang H., An H., Zhao Y. (2023). Accuracy and modification of the STOP-bang questionnaire for screening patients with obstructive sleep apnea in China. J. Sleep Res..

