# Supplementary material for: Longitudinal Association Between Menopausal Transition and Obstructive Sleep Apnea with Effect Modification by Salt Intake: A Prospective Cohort Study
Source: Nutrients. 2025 Nov 19;17(22):3612. doi: 10.3390/nu17223612 (PMC12655854; doi:10.3390/nu17223612)
Supplement: Supplementary file 1 [file nutrients-17-03612-s001.zip › nutrients-3956497-supplementary.pdf]

Supplementary Figures

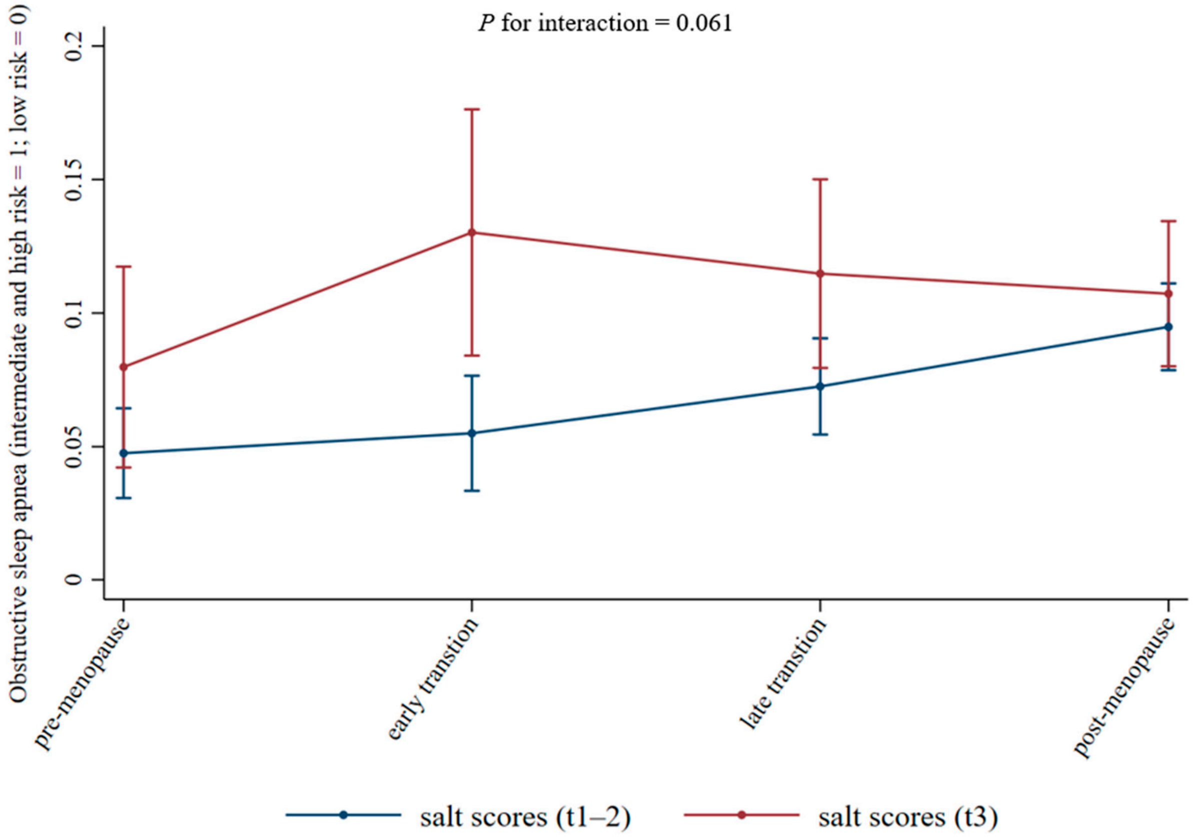

Supplementary Figure S1. Sensitivity analysis of OSA risk trends across menopausal transition stages by salt intake (independent correlation). Error bars represent 95% confidence intervals.

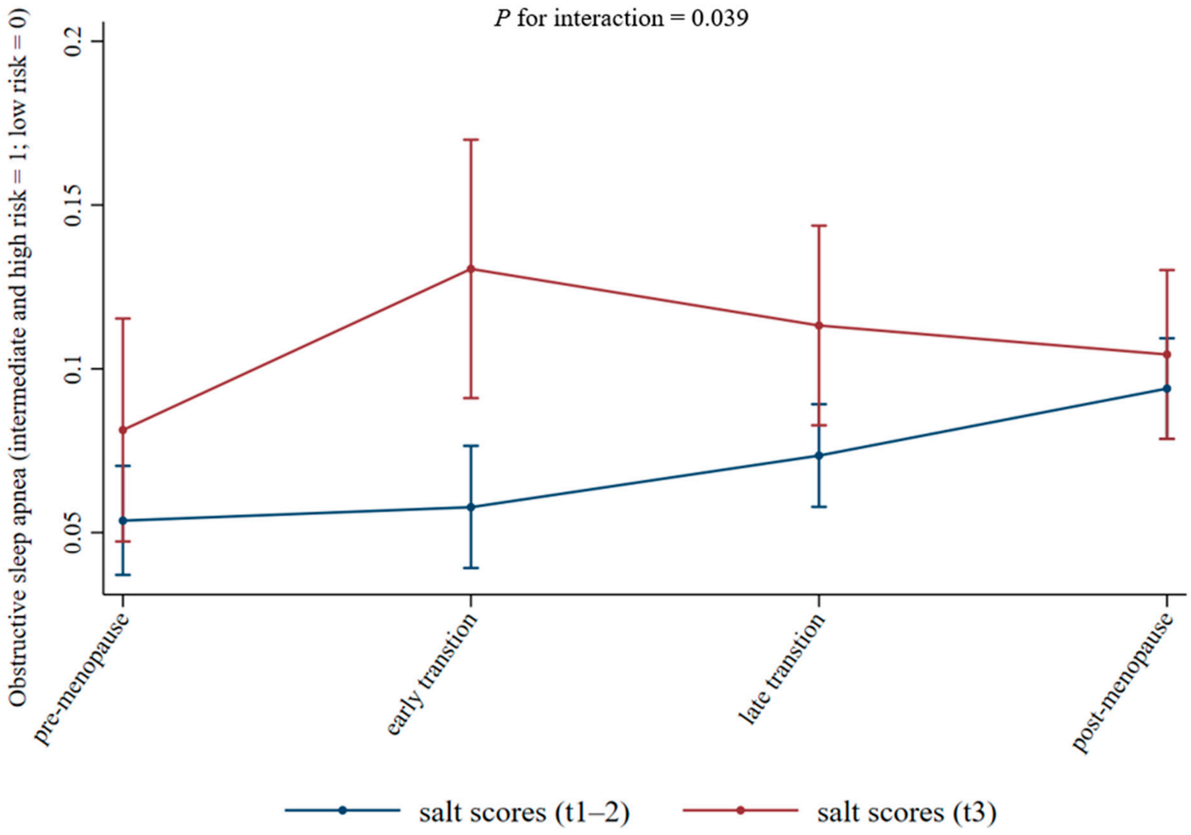

Supplementary Figure S2. Sensitivity analysis of OSA risk trends across menopausal transition stages by salt intake, incorporating inverse probability weighting (IPW). Error bars represent 95% confidence intervals.

Supplementary Tables

Supplementary Table S1. Association between menopausal transition and salt intake and OSA

Variable	Coefficient (95% CI)	*p*-value
**Time-varying age (year)**	0.21 (0.17–0.25)	<0.001
**Menopausal transitions**		
Pre-menopause	Ref	
Early transition	0.30 (-0.10–0.69)	0.139
Late transition	0.41 (0.05–0.78)	0.026
Post-menopause	0.61 (0.20–1.02)	0.004
**Salt intake scores**		
Tertiles 1–2	Ref	
Tertile 3	0.41 (0.18–0.65)	<0.001

The model was adjusted for smoking status (never smoker, ever smoker, unknown), alcohol consumption (<10 g/day, ≥10 g/day, unknown), physical activity level (inactive, moderately active, HEPA, unknown), BMI (continuous), parity (nulliparous, parous, unknown), marital status (unmarried, married/cohabiting, divorced/separated/widowed, unknown), and education level (≤high school graduate, ≥college graduate, unknown) as time-varying covariates; and for age at menarche (<12, 12−13, 14−16, ≥17 years, unknown) as a fixed covariate, with clustering according to individual identifiers.

Abbreviations: BMI, body mass index; CI, confidence interval; HEPA, health-enhancing physical activity; OSA, obstructive sleep apnea

Supplementary Table S2. Associations between OSA and covariates

Variable	Coefficient (95% CI)	*p*-value
**BMI**[1]	0.27 (0.23–0.31)	<0.001
**Smoking**		
Never	Ref	
Currently/formerly	0.16 (-0.23–0.55)	0.427
**Alcohol consumption**		
<10 g/day	Ref	
≥10 g/day	0.22 (-0.30–0.75)	0.405
**Physical activity**		
Inactivity	Ref	
Moderate activity	-0.09 (-0.32–0.13)	0.425
HEPA	0.07 (-0.22–0.36)	0.633
**Parity**		
Nulliparous	Ref	
Parous	-0.19 (-0.83–0.45)	0.563
**Marital status**		
Married/cohabitating	Ref	
Unmarried	-0.70 (-1.68–0.29)	0.164
Divorced/separated/widowed	-0.03 (-0.69–0.63)	0.927
**Education**		
≤High school	Ref	
≥College	-0.20 (-0.52–0.11)	0.208
**Age at menarche**		
<12 years old	Ref	
12–13 years old	0.64 (-0.15–1.44)	0.114
14–16 years old	0.71 (-0.08–1.50)	0.076
≥17 years old	0.55 (-0.44–1.55)	0.277

[1]BMI was analyzed as a continuous variable (kg/m^2)

All covariates are time-variant, except for age at menarche

Abbreviations: BMI, body mass index; CI, confidence interval; HEPA, health-enhancing physical activity; OSA, obstructive sleep apnea
